# *Neurotransmitter Transporter-Like*: A Male Germline-specific SLC6 Transporter Required for *Drosophila* Spermiogenesis

**DOI:** 10.1371/journal.pone.0016275

**Published:** 2011-01-27

**Authors:** Nabanita Chatterjee, Janet Rollins, Anthony P. Mahowald, Christopher Bazinet

**Affiliations:** 1 Department of Biological Sciences, St. John's University, Jamaica, New York, United States of America; 2 Department of Molecular Genetics and Cell Biology, University of Chicago, Chicago, Illinois, United States of America; French National Centre for Scientific Research - Université Aix-Marseille, France

## Abstract

The SLC6 class of membrane transporters, known primarily as neurotransmitter transporters, is increasingly appreciated for its roles in nutritional uptake of amino acids and other developmentally specific functions. A *Drosophila* SLC6 gene, *Neurotransmitter transporter-like (Ntl),* is expressed only in the male germline. Mobilization of a transposon inserted near the 3′ end of the *Ntl* coding region yields male-sterile mutants defining a single complementation group. Germline transformation with *Ntl* cDNAs under control of male germline-specific control elements restores *Ntl/Ntl* homozygotes to normal fertility, indicating that *Ntl* is required only in the germ cells. In mutant males, sperm morphogenesis appears normal, with elongated, individualized and coiled spermiogenic cysts accumulating at the base of the testes. However, no sperm are transferred to the seminal vesicle. The level of polyglycylation of *Ntl* mutant sperm tubulin appears to be significantly lower than that of wild type controls. Glycine transporters are the most closely related SLC6 transporters to *Ntl,* suggesting that *Ntl* functions as a glycine transporter in developing sperm, where augmentation of the cytosolic pool of glycine may be required for the polyglycylation of the massive amounts of tubulin in the fly's giant sperm. The male-sterile phenotype of *Ntl* mutants may provide a powerful genetic system for studying the function of an SLC6 transporter family in a model organism.

## Introduction

The ease with which *Drosophila* male-sterile mutations are isolated indicates that a large number of genes must contribute information required for spermatogenesis [1], [2], [3]. This implies great complexity (as defined by Adami [4]) in some aspect(s) of spermiogenesis, borne out by microarray analysis demonstrating that more genes are transcribed in the testis than in any other organ or tissue of *Drosophila* [5]–[6]. Mammalian tissue transcriptomes show the same pattern, perhaps more strongly [7].

Guo et al [8], reported greater similarity between brain and testis transcriptomes than those of any other mammalian tissues. In *Drosophila,* numerous mutations identified in genetic screens for behavioral abnormalities, or through targeting of fly homologues of human genes implicated in neurological disease states have yielded male-sterile mutations. The former include *pushover (poe)* [9], *tilB* [10], *unc* [11], *yuri* [12], and *gish* [13], [14], [15] while the latter include *Dmfr1,* the *Drosophila* homologue of the Fragile X protein, dfxr [16]. The fly homologues of two human genes implicated in the etiology of Parkinson's disease, *parkin* [17], [18] and *pink* [19], are also required for sperm morphogenesis. This apparent “crosstalk” between the CNS and testis transcriptomes is frequently noted anecdotally in the literature, but it remains to be understood what, if any, biological significance such observations indicate.

The highly-conserved Neurotransmitter Sodium Symporter (NSS) family includes serotonin, dopamine, and GABA transporters, as well as amino acid and osmolyte transporters in both prokaryotes and eukaryotes [20], [21], [22], [23]. These transporters, also known as the SLC6 (Solute Carrier 6) family and SNF (Sodium Neurotransmitter Symporter Family), comprise the largest subfamily of neurotransmitter transporters. NSS family members are an ancient group of highly conserved integral membrane proteins with characteristic twelve transmembrane pass domains. They are crucial for transporting neurotransmitters, amino acids and osmolytes across the plasma membrane. They are generally referred to as the neurotransmitter transporter family because the classical members were transporters for GABA, norephinephrine, serotonin, and dopamine. However, additional family members are now known to mediate the cellular uptake of amino acids and their derivatives in a number of tissues [20], [21].

The NSS/SLC6 family is notable for the number of psychological/neurological disorders that have been linked to changes in their activities, and for the remarkable number of pharmacological agents that target them, including psychostimulants, antidepressants, and neurotoxins [23], [24]. The primary molecular targets of cocaine, amphetamines, fluoxetine (Prozac), citalopram (Celexa), paroxetine (Paxil), sertraline (Zoloft), MDMA (Ecstasy), methylphenidate (Ritalin), tricyclic antidepressants, nomifesine and bupropion are neurotransmitter transporters of this class. The large number of natural products and pharmacologically-derived compounds that act through these transporters indicates that both evolution and medicinal chemistry have converged on them as strategic nodes for the regulation of behavioral pathways.

Despite their significance in mammalian physiology, relatively little is known about these transporters in the model organism *Drosophila melanogaster*. Thus far five out of a total twenty two predicted *Drosophila* members of this family have been characterized, including the serotonin transporter homolog (*SerT*) [25], [26], dopamine transporter (*DAT*) [27], and a probable carcinine transporter encoded by the *inebriated* (*ine*) gene [28], [29], [30]. Additional *Drosophila* family members characterized are *bloated tubules* (*blot*) [31], required early in embryogenesis, and DmNAAT1, the first nutrient amino acid transporter identified in *Drosophila* [32].

Previous studies by R. Dörig and D. Bigler [33], [34] had identified a *Drosophila* gene encoding a testis-specific member of the NSS family, which they named ***N*** *eurotransmitter* ***t*** *ransporter-* ***l*** *ike (Ntl).* Using an *Ntl-LacZ* fusion construct, Bigler showed that *Ntl* was expressed in developing sperm, and that the cellular distribution of *Ntl* protein exhibited dynamic reorganization during spermiogenesis. Here we report the isolation of male-sterile mutants in the *Drosophila* gene *Neurotransmitter transporter-like (Ntl),* a member of the NSS/SLC6 family, and confirm that the gene is expressed only in male germline cells. Sperm produced by individuals homozygous for a null mutation in *Ntl* are morphologically mature, but are immotile and fail to be transferred to the seminal vesicle. The massive posttranslational glycylation of sperm tubulin observed late in spermiogenesis is consistently reduced in *Ntl* mutants. This is in accord with the suggestion that glycylation is important for stability and/or motility of microtubule-based machinery [35], [36], [37], [38]. The strong similarity of *Ntl* to known glycine transporters suggests that it functions to augment the cytosolic glycine pool in male germ cells.

Because *Ntl* mutants are perfectly viable but completely male-sterile, they present an outstanding opportunity for the study of an NSS transporter function in a well-defined but complex cellular morphogenesis pathway. The very high conservation of *Ntl* homologues in mosquito vectors and the highly developed pharmacology of the NSS/SLC6 family also suggest a potential route to vector population control.

## Materials and Methods

### Fly Husbandry

Flies were raised on standard cornmeal molasses agar at 25°C [39]. Unless otherwise mentioned, all stocks were from the Bloomington Stock Center. Males were tested for fertility by mating in groups of 4–5 with an equal number of virgin females. Generally, *w^+^* or *y^+^* males were mated with *yw* females, with the recovery of *y^+^* or *w^+^*daughters in the F1 generation confirming fertility. Genetic constructions were carried out using standard Drosophila genetics as in Greenspan [40].

For fluorescence microscopy of sperm individualization, wild type and mutant freshly eclosed *Drosophila* males carrying dj-GFP were withheld from females for eight days before dissecting their testes to check for dj-GFP expression. Lines expressing β tubulin-GFP were a kind gift from S. Goto via Karen Hales.

### Generation of Ntl mutants

The Ntl transcript/CDR is in the 28C region on the 2L arm of the *Drosophila* chromosome. The P{EPgy2}*^Ey05549^* transposon insertion was generated by the Drosophila Genome P element disruption project [41]. The transposon was mobilized by crossing the chromosome carrying it to the stable Δ2–3 source of transposase [42]. Chromosomes that lost the *w^+^* and/or *y^+^* markers carried by the P element were recovered using standard *Drosophila* genetics, then screened for new male-sterile mutations expected from the deletions produced by imprecise excision of the P element [43].

### RNA isolation and RT-PCR

Total RNA was isolated using TRI reagent (Sigma) according to the manufacturer's recommendations. RNA was extracted from males, females, ovaries, testes, heads and carcasses and concentration was determined by measuring its absorbance at 260 nm. 1–2 µg of total RNA was used after normalizing for all samples.

Two-step RT-PCR was performed using SuperScript ™III Reverse Transcriptase (Invitrogen) according to the manufacturer's recommendations. During first strand synthesis incubation with gene specific primers were carried out at 55°C for 60 min. The Thermocycler (MJ Research Gradient cycler) was programmed as follows: 95°C for 5 min followed by the amplification steps of 94°C for 1 min, 57°C for 1 min, 72°C for 1 min. 30 cycles of PCR was run for all samples followed by 10 min at 72°C and held at 4°C overnight. *Ntl* gene specific primers were designed to span an intron/exon boundary to avoid genomic DNA amplification in case of contamination.

*Ntl* primers amplified a 629 bp fragment, while *rp49* control primers amplified a 405 bp fragment. Primers for this and all subsequent PCR-based molecular biology are specified in Table 1.

**Table 1:** Primers.

Primer pair	Upstream (distal)	Downstream (proximal)	Coordinates 1 = 1^st^ base of AT20383
*Ntl* RT-PCR	GTCGGTTGGCAAGGTTGTGT	ACATGCCGCCATTTGTGCAC	
*Rp49* RT-PCR	AGCGCACCAAGCACTTCATC	GTGCGCTTGTTCGATCCGT	
Interval A1	GTGACAATGGCGAATAATCA	AGAAACCGACAAAGAAAACG	1–682
Interval A2	GTGACAATGGCGAATAATCA	TATGCCAAAGAACATCTGGA	1–1648
Interval B	GCAAAATAGTAGTGATGTGTTC	TATGCCAAAGAACATCTGGA	1172–1648
Interval C	ACCTCAAAGTCTGGGCCGATG	TGAAGAATAAACATGCCGCCC	1600–2280
Interval D	ATATTGGATGAGTGGTATTG	GTCCTTATGAATACAATGCTAG	2107–2554
Interval E	TTTATGCTCGGAAAACGGCC	AGACATAAATCCCGGCATTCG	2390–2795
Interval F	CTGGGCCAGTTGTTTTACAT	TCTTTGTACCCTTGTTCCAA	2603–2913
Genomic fragment ligated into pCaSpeR4	GACGATGGTACCCCCGCTCCGTGTCCTTCATCGTGACTCCGAAGA	GACGATGGTACCGGATCTATGGCCAAAAACGCTTCCAGCGGG	
cDNA site-directed mutagenesis	TTGGCCGGATTTGTGGTCTTCTCCGTGTTGG	CCAACACGGAGAAGACCACAAATCCGGCCAA	
Not1 linker primers for pTMR cloning of *Ntl* cDNA	GCATATATATTGCGGCCGCGTGACAATGGCGAATAATCAGCCCCCGACAACG	GCGCGCGCGCGGCGGCCGCGGATTAACAATATATTAACTTAAAT	

### Deletion PCR

Genomic DNA was isolated from males according to the Berkeley Drosophila Genome Project's protocol (http://www.fruitfly.org/about/methods/inverse.pcr.html). 6 pairs of genome specific primers were used to span the entire coding sequence of *Ntl*. The thermocycler (MJ Research gradient cycler) was programmed as follows: 95°C for 5 min followed by the 30 cycles of: 94°C for 1 min, 57°C for 1 min, 72°C for 1 min. After a final 10′ at 72°C, samples were held at 4°C until gel analysis.

### Plasmid Constructions

To generate pCaSpeR4-Ntl; *Ntl* genomic DNA from with 2.7 kbs upstream including Ntl promoter site was amplified using TaKaRa LA PCR kit ver 2.1 from a BAC clone of 2L arm of the Drosophila chromosome BACRO9A04, using *Kpn*I tagged primers, cloned into Psc-A vector using StrataClone PCR cloning kit (Stratagene). The 6003 bp fragment was recovered by digesting with *Kpn*I, gel purified and ligated into pCaSpeR4 vector (DGRC).

To generate pTMR-Ntl, full-length cDNA clone AT20383 was obtained from the Drosophila Genome Research Center. Comparison of the AT20383 sequence with the predicted sequence in the NCBI database suggested a single base pair deletion in AT20383 leading to a frameshift mutation and premature termination of the *Ntl* reading frame. The missing “G” was inserted at position 961 of AT20383 by site-directed mutagenesis (QuickChange, Stratagene) and the change confirmed by sequencing. The sequences of other cDNAs since deposited in the databases confirm that the missing base was a cloning artifact. Long Range PCR was done on the repaired clone (AT20383) using PfuTurbo DNA Polymerase (Stratagene) to attach *Not*I sites at either end for ligating into pTMR vector [44] (a gift from Dr. Ming Guo). Orientation of Ntl cDNA with respect to the β-tubulin promoter was checked by *Eco*RI digestion.

Primers were designed using the Vector NTI software. Primers for SDM were designed using the QuikChange Primer Design Program (http://stratagene.com/sdmdesigner/).

All DNA cloning procedures were carried out according to standard methods.

P element transformation constructs were injected by BestGene Inc., Chino Hills, CA.

### Phalloidin assay for Individualization Complex

Testes were dissected from freshly closed males in 1X phosphate-buffered saline (PBS), transferred to a drop of fixative (1.8% formaldehyde in PBT) on poly-lysine coated slides for 10 minutes, then rinsed three times with PBT (0.1% Triton X in PBS), blocked in 1.5% BSA in PBT for 20 min and stained with 1 µg/ml TRITC-phalloidin (Sigma Chemical, St Louis, MO) in PBT for 1 hour at room temperature. Slides were then rinsed three times in PBS and mounted in 10%(w/v) Mowiol (Calbiochem, San Diego,CA) 4–88, 25% glycerol, 100 mM Tris-HCl, pH 8.5. Slides were stored overnight at 4°C before imaging. Fluorescence images were captured by confocal microscopy (Leica TCS-2, Exton, PA).

### Phase and fluorescence squashes

*Don-juan*-GFP (dj-GFP) was expressed in the testes by crossing a dj-GFP/TM3, *Sb* line into *Ntl*/SM6a mutant background to generate a stock. Testes were dissected from 0–1 day old young male Drosophila (unless mentioned otherwise) in 1X PBS buffer and gently squashed with a cover slip before taking fluorescence and phase images with Leica DM 4500B.

### Electron Microscopy

Testes were dissected from 1–3 day-old males, fixed and embedded in Epon/Araldite. Thin sections were stained and photographed by TEM as previously described [45].

### Protein Electrophoresis and Immunoblot:

Samples were prepared from male fly abdomens for each genotype. The males were withheld from females for 4–6 days. Dissection of the testes sometimes leads to loss of seminal vesicle, which contain significant amounts of glycylated tubulin in wildtype (*yw*) flies. Therefore, whole abdomens were used to prepare protein samples. Six fly abdomens worth of protein (∼4–5 µg) were loaded in each lane.

Abdomens were dissected in 1X PBS, ground in 2X Laemmli buffer(Laemmeli et al.,1970) vortexed and boiled for 5 minutes. Samples were then spun at 15,800 g for 5 minutes, and supernatants were separated in a 15% SDS-Polyacrylamide gel and transferred to a PVDF membrane (Amersham, GE Healthcare, United Kingdom) using a Trans-Blot Semi-Dry transfer apparatus (BioRad, U.S.A).

Membranes were incubated with Poly-G(1∶10,000), (gift of M.Gorovsky) and anti-α-tubulin antibody (1∶5000), (DM1A, Sigma).Protein bands were visualized with HRP-labelled anti rabbit or anti-mouse (1∶10,000; both courtesy of Rachel Zufferey) followed by detection with ECL immunoblot detection kit (Pierce, U.S.A). Loading control was α-tubulin from duplicate lanes.

All statistical analyses were done using Microsoft Excel. The average values of relative intensity (poly-G intensity/anti-tubulin) were plotted for each genotype. Intensities were calculated using Image J software (Rasband, W.S., ImageJ, U. S. National Institutes of Health, Bethesda, Maryland, USA, http://rsb.info.nih.gov/ij/, 1997–2009).

## Results

### Amino acid sequence alignment of Ntl with SLC6 transporters

**Figure 1:**
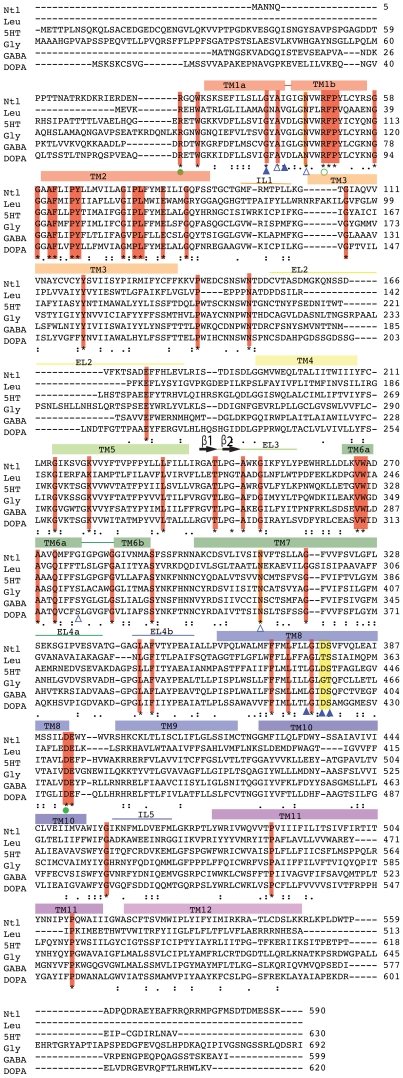
Amino acid alignment of Ntl with human and *A.aeolicus* SLC6 transporters. Amino acid sequence alignment of *Drosophila melanogaster* Ntl (Ntl; NP_609135.1) with A.aeolicus Leu T_Aa_ (Leu; NP_214423), human homologues for Serotonin (5HT;P31645), Glycine (Gly, I57956), GABA (GABA; P30531), Dopamine (DOPA; Q01959) using Clustal W alignment [http://www.ebi.ac.uk/Tools/clustalw2/index.html]. Strictly conserved residues are highlighted in red; α coils and β sheets are depicted as blocks and arrows respectively. Open and filled green circles represent putative cationic gates at extra and intra cellular surfaces (EL and IL) respectively. Open and filled blue triangles indicate sites that interact with sodium ions in the *LeuA* structure. Tyrosine in TM3 is a critical residue present in Ntl, which is indispensable for substrate binding and transport. Adapted from Yamashita *et al*, 2005.

Clustal W alignment of the predicted *Ntl* protein sequence with known members of the SLC6 family is presented in Figure 1. All of the amino acid residues considered critical for transporter function [46] and conserved in the other 4 members included in this alignment are conserved in the predicted Ntl protein. The TMpred algorithm [47] predicts a 12 transmembrane domains for *Ntl,* characteristic of the SNF family (data not shown).

### Generation of mutants

**Figure 2:**
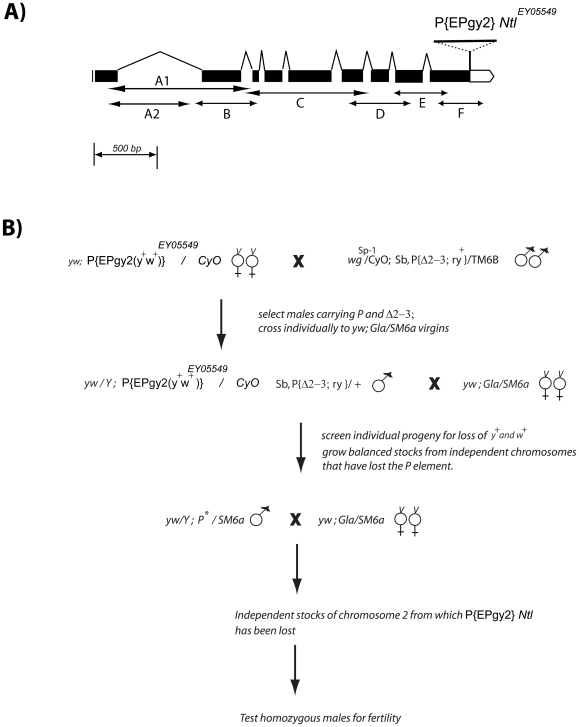
Generation of *Ntl* mutants. A) *Ntl* transcript/CDR from 28C on the left arm of chromosome 2. P element is inserted at the very end of the protein coding region. B) Scheme for generation of *Ntl* deletion mutations by imprecise excision of the P element.

*Ntl* mutants were generated by mobilizing a P element (P {EPgy2} CG7075 ^EY05549^) at the 3′ end of the *Ntl* gene by crossing it into genetic background in which transposase is constitutively expressed in all tissues [48]. By standard fly genetics, chromosomes which had lost the *yellow* ^+^ (*y^+^*) and/or *white^+^ (w^+^)* markers associated with the P element were recovered and established in balanced genetic stocks. P element excision is imprecise and deletions of varying size spanning the insertion site or extending from the insertion site in either direction are often recovered at significant frequencies [43]. A schematic of the *Ntl* locus showing the starting insertion, the mating scheme used to mobilize the transposon and identify new male-sterile mutations, and the location of primer pairs used to assay for deletions in the resulting male-sterile stocks is shown in Figure 2.

Of 150 chromosomes that were observed to have lost the P element, 8 were found to carry new male sterile mutations defining a single complementation group. Several homozygous lethal mutations were also recovered. These fail to complement the male-sterile alleles and are likely to be larger deletions including a functionally essential portion of the *Ntl* gene and extending further into one or more essential genes.

### Deletion PCR analysis and Rescue by Germline Transformation

The mutant alleles were screened with overlapping gene specific primers (Materials and Methods and Figure 2). We used the genomic DNA from homozygous male carrying each of the 8 male sterile alleles to probe for changes in the chromosome structure by PCR. As expected, PCR fragments amplified with primer pairs spanning the site of the transposon insertion or located in the 3′ ∼half of the gene were more frequently missing or altered in size when the male-sterile mutant DNA was used as template for amplification (Table 2). The results of this approach indicate that *Ntl* ^129A^ is a null mutant, as none of the primer pairs tested using *Ntl^129A^/Ntl^129A^* DNA as template yielded a PCR fragment corresponding to that obtained from the wild-type control (Table 2). Additionally, the phenotypes of *Ntl^129A^* over the deficiencies *BSC324* and *Bsc192*, which extend through *Ntl* from the distal and proximal sides respectively, are indistinguishable from the phenotype of *Ntl^129A^*/*Ntl^129A^* homozygotes. For phenotypic analysis, the *Ntl^129A^* allele has therefore been used, and the phenotype observed is assumed to be the true null phenotype.

**Table 2:** Deletion analysis of *Ntl* alleles.

Alleles	1–682 (A1)	1–1648 (A2)	1172–1648 (B)	1600–2280 (C)	2107–2554 (D)	2390–2795 (E)	2603–2913 (F)
Wild type	+	+	+	+	+	+	+
140B	+	−	Disrupted	NSB a	Disrupted	Disrupted	+
25B	+	+	+	+	+	+	−
181B	+	+	+	+	+	+	+
144A	+	−	−	NSB	−	−	Disrupted
69B	+	+	+	+	+	+	−
129A	−	−	−	−	−	−	−
172B	+	+	+	+	+	+	Disrupted
54A2	+	−	−	−	−	−	−

Numbers correspond to coordinates of Ntl genomic sequence.

+/− Refer to presence or absence of the band respectively.

a Non specific bands.

**Figure 3:**
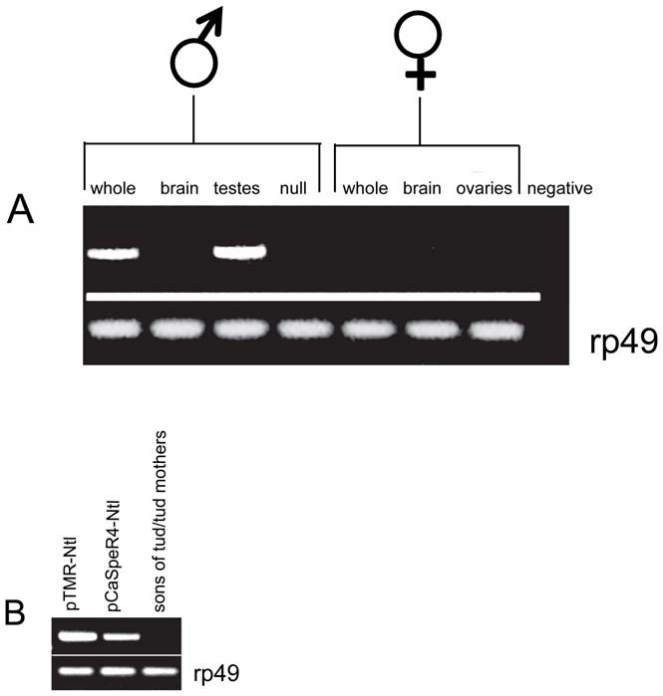
*Ntl* expression is testis specific and limited to the germline. A) From the left: *Ntl* RT-PCR products from wt whole males, wt male heads, wt male testis, 129A whole male; *Ntl* RT-PCR products from wt whole females, wt female heads and wt female ovaries. The last lane is a negative control without RNA. B) *Ntl* RT-PCR products from *Ntl/Ntl* mutant males carrying a pTMR-*Ntl* cDNA construct (Lane 1; *yw; Ntl^129A^/Ntl^129A^; pTMR-Ntl1/TM3,Sb*) and a genomic pCaSpeR4 construct (Lane 2; *yw; Ntl^129A^/Ntl^129A^*; pCaSpeR 4- Ntl1/*TM3,Sb*). Lane 3: *Ntl* RT-PCR product from testes of male offspring of *tud/tud* females, which lack germ cells.

To confirm that the male sterile phenotype in our mutants is caused solely due to disruption of *Ntl*, we generated P-element-based constructs for germline transformation with CG7075/*Ntl*. Towards this end we obtained a full length cDNA clone of CG7075 (AT20383) from the Drosophila Genomics Research Center, modified it as described in Materials and Methods, and cloned it downstream of the β2T-tubulin transcriptional control sequences in the *Drosophila* transformation vector pTMR [44]. This provides for strong germ cell-specific transcription [49], [50]. All six 3^rd^ chromosome pTMR insertion lines rescued the *Ntl* mutant male sterile phenotype. Results from one of the rescued lines are presented in figure 3B. A pCaSpeR 4 genomic construct extending from 2.7 kb upstream of the *Ntl* transcript to 519 bp beyond its 3′end also rescued the *Ntl* mutant phenotype (4 lines; all rescued). Rescue by the genomic construct indicates that all cis-acting control sequences required for functional *Ntl* expression lie within 2.7 kb upstream (distal) of the gene. Results from one of the rescued lines are also presented in figure 3B. *Ntl^129A^/Cy* stocks carrying the P insertions are stable, with fertility of homozygous mutant males segregating consistently with the *w^+^* marker of the P element. The male-sterile complementation group recovered after mobilization of the P element therefore corresponds to the *Ntl* gene.

### Specificity of *Ntl* expression:

RT-PCR analysis confirms that *Ntl* expression is male-specific and limited to the testes (Fig3A). There is no expression in females nor in the adult heads of either sex. This is in agreement with Dörig (1991) [33] and Thimgan *et al* [51]. To determine if *Ntl* expression is limited to the germline, we performed RT-PCR on RNA extracted from male progeny of *tud* ^−/−^ mothers. These males have no germ cells [45]. Since *Ntl* transcript was not detected in their testes, this strongly argues that the *Ntl* expression is limited to the male germline. (Figure 3B). The rescue of *Ntl* mutants by transgene expression under control of the germline-specific β2-tubulin promoter further confirms that germline expression alone is sufficient for *Ntl* function.

### Spermiogenic-defective phenotype of *Ntl* mutants

**Figure 4:**
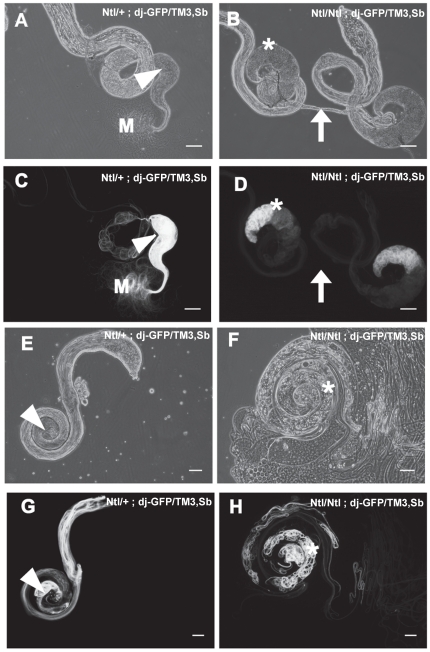
*Ntl* mutant sperm are immotile and are not transferred into seminal vesicles. Panels A, B, E, and F: Phase contrast images of testes from *Ntl^+^* (A, E) and *Ntl^-^* (B, F) males. The major phenotypic feature of the mutants is the accumulation of coiled cysts at the base of the testis (asterisks), and the empty/shrunken state of the seminal vesicle (arrows). Panels, C, D, G, and H: *don juan-*GFP fluorescence images corresponding to phase images immediately above them, showing the disposition of elongated cysts and mature sperm in the testis and seminal vesicle. Note the complete absence of fluorescence from the seminal vesicle of *Ntl/Ntl* mutants (arrows) compared to the accumulated fluorescence in wild type seminal vesicles (arrowheads), and accumulation of coiled cysts in the base of the mutant testes (asterisks). In panels A and C, the letter M demotes dense masses of mature motile sperm which is not seen in the mutants. Left hand panels: wild type (*Ntl/+*); right hand panels: *Ntl* ^129A^/*Ntl* ^129A^ mutants. Bars, 20 µm.

Early stages of sperm development appear normal by standard phase-contrast microscopy of testis squash preparations (data not shown). In a genetic background expressing *don juan-GFP* [52], which labels elongated sperm and spermiogenic cysts, *Ntl/Ntl* males produce elongated spermiogenic cysts (Figure 4) but we have never detected mature sperm in the seminal vesicle (Fig. 4, B and D, arrows). In contrast, the WT control seminal vesicle is filled with mature sperm (Fig. 4, A, C, E and G, arrow heads). No motile sperm are seen in *Ntl/Ntl* squash preparations, unlike in the WT control where dense masses of mature motile sperm are evident (Fig. 4, A and C, letter M). Instead, an extensive mass of coiled sperm bundles accumulates at the base of *Ntl* mutant testes (Fig. 4 B, D, F, H asterisks).

**Figure 5:**
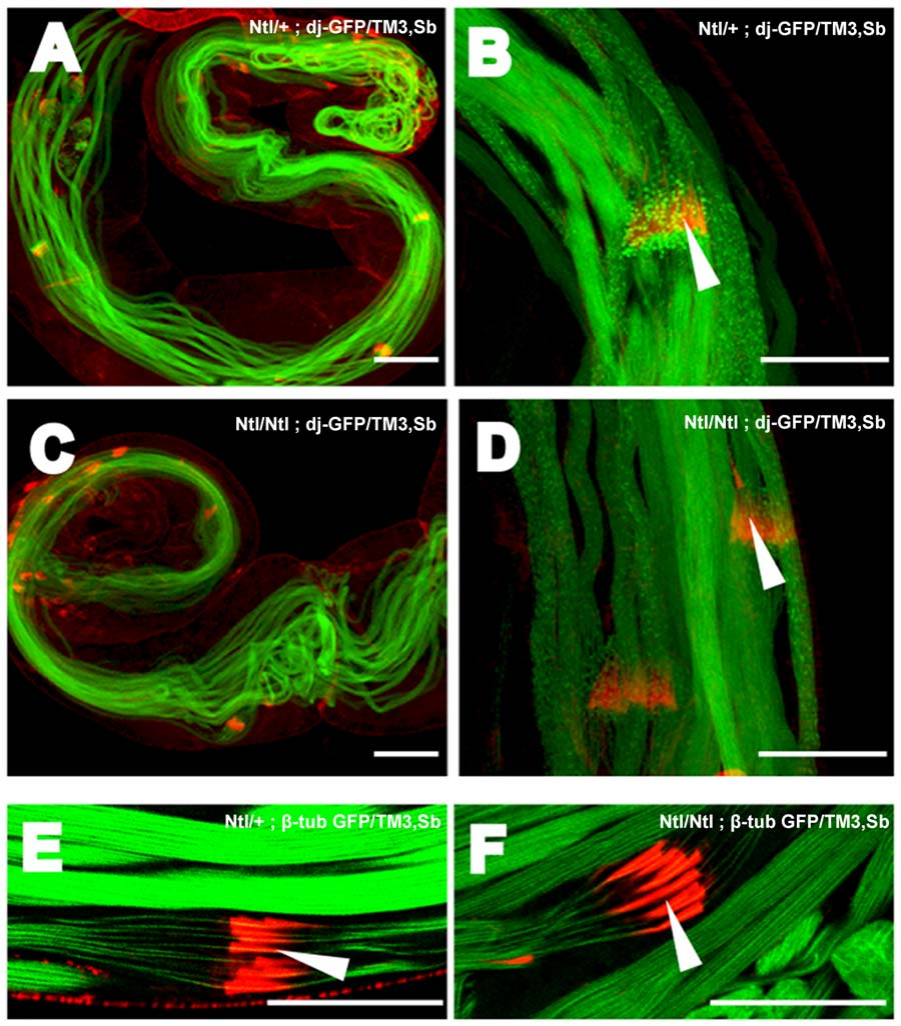
Individualization in *Ntl* mutants. A, B: wt testis expressing *don juan*-GFP counterstained with TRITC phalloidin to visualize the actin cone-based individualization complexes. Arrowhead marks the actin cones of the complex. C, D: *Ntl* mutant testis preparations expressing *dj-*GFP, counterstained with TRITC-phalloidin. Formation and movement of actin cones/individualization complex along the mutant cysts appears normal. E, F: TRITC-phalloidin staining of W.T. (*Ntl/+*) (E) and *Ntl* mutant (F) cysts from males expressing β*Tub-*GFP. Bars, 20 um.

Presumably because of the great physical complexity of the sperm individualization process [53], [54], a large proportion of male-sterile mutations produce elongated cysts that fail to be matured into individual sperm. In most of these cases, the individualization complex either fails to form, does not progress, or breaks down during its transit along the length of the cyst [55]. When mutant *Drosophila* testes carrying dj-GFP were counterstained with TRITC conjugated phalloidin, we observed normal formation and movement of the actin cones of the individualization complex (Figure 5). Waste bag deposition in the distal end of the testis also appeared normal (not shown), further indicating that the individualization complex was successfully traversing the entire length of the cysts. When we counterstained mutant *Drosophila* testes carrying β-tubulin-GFP, with TRITC-phalloidin, we observed that the cones formed perfectly around the axonemes and IC progression was comparable to wild type (Figure 5, E and F).

**Figure 6:**
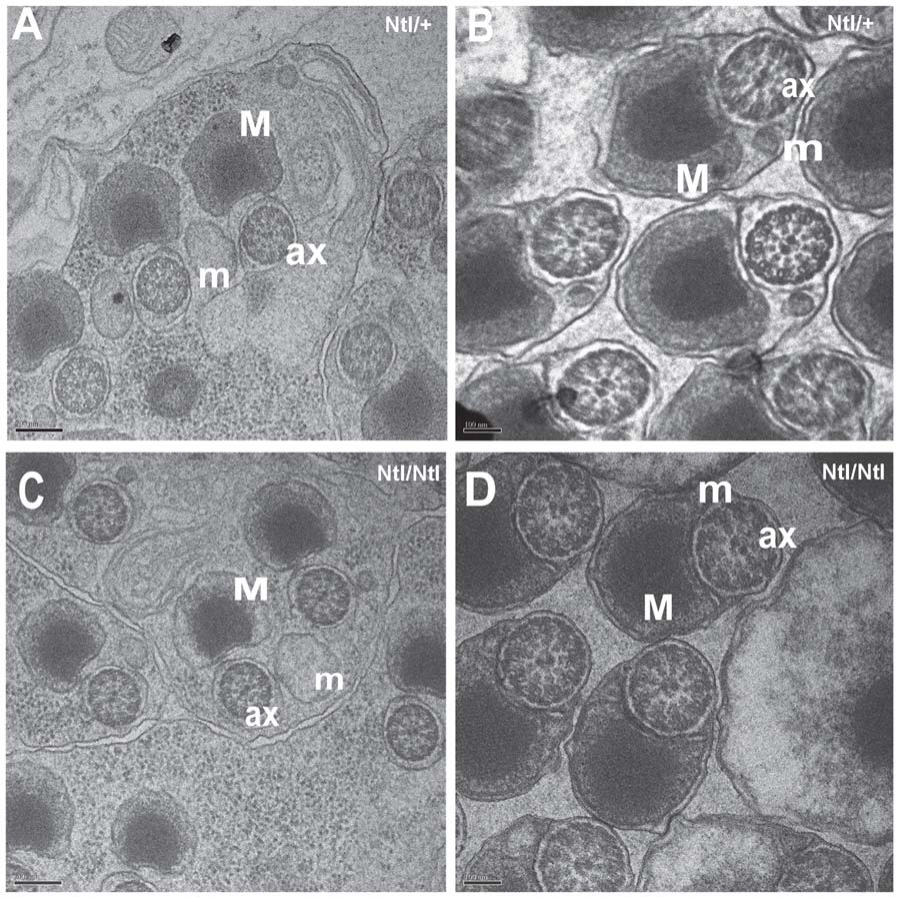
Ultrastructure of *Ntl* mutant spermiogenesis. A, B: TEM of cross-sections through wild-type pre-individualized and post-individualized cyst respectively. C and D: cross-sections through pre-individualized and individualized cyst of *Ntl/Ntl* mutant cyst. No obvious differences between the mutant and the wild type phenotype were observed at this level of resolution. ax, axoneme; M, major mitochondrial derivative; m, minor mitochondrial derivative. Bars, 200 um (A, C); 100 um (B, D).

Transmission electron micrographs of cross-sections through pre and post-individualized cysts (through the base of the testes) do not show any obvious defect in the individualization process nor the axoneme structure (Figure 6). Examination of the content of *Ntl* mutant cysts released from the base of the testis and examined by phase microscopy revealed the very consistent and smooth profile of wild-type individualized sperm (data not shown). In summary, morphogenesis of *ntl* mutant sperm appears quite normal at both optical and ultrastructural resolution, but the apparently immobile end products of spermiogenesis are not transferred to the seminal vesicle.

### Reduction of tubulin polyglycylation in *Ntl* mutants

**Figure 7:**
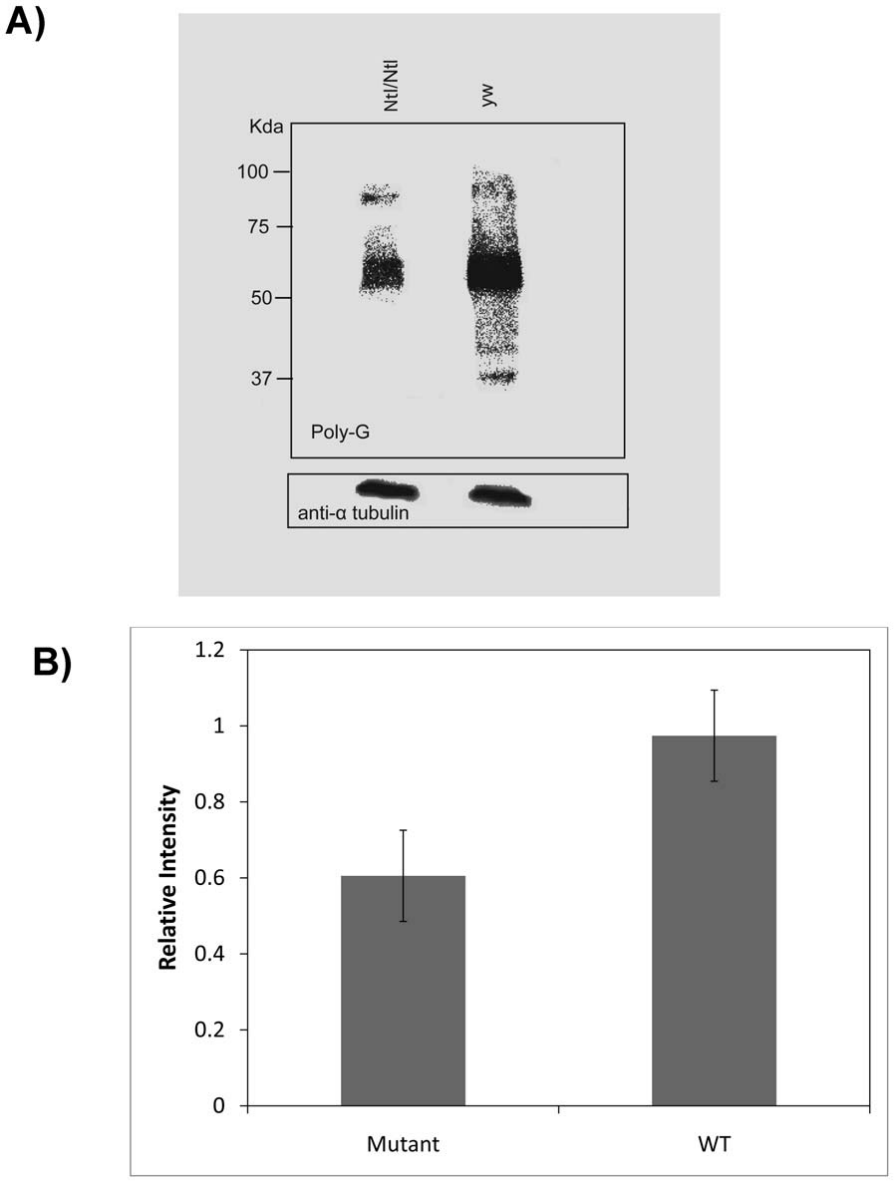
Polyglycylation of tubulin is partially decreased in mutant testes. A)Western Blot of *Ntl/Ntl* male abdomens compared to +/+ *(yw)* probed with anti Poly-G antiserum. B) Quantitation of 6 independent replicates of the Western analysis of panel A. Bar, standard error of the mean. N = 6.

The tubulin of *Drosophila* sperm axonemes and many other eukaryotic cilia, has been shown to be modified via polyglycylation, the addition of glycine chains through the γ-carboxyl group of glutamate residues near the C-terminus of tubulin [35], [36], [56]. Recently, it has been demonstrated that RNAi depletion of the gene encoding the *Drosophila* glycylase, dmTTLL3B, results in male sterility [37]. Because the predicted amino acid sequence of *Ntl* is most similar to known human glycine transporter GLYT2 (P = 9 e^−134^ in BLASTP against nonredundant database with default parameters), we assayed *Ntl* mutant males for tubulin glycylation using anti Poly-G antiserum [57]. Quantitation by scanning the results from 6 independent experiments, using α-tubulin as a loading control showed an average of 40% reduction in poly-G signal in the mutant samples relative to wild-type (Figure 7). Although we observed considerable variation in the Western analysis experiments, the glycylation of the *Ntl* mutant was never observed to be higher than that of the wild-type. The semi-quantitative nature of this assay and variation between individuals sampled for the experiments could account for this variation. More quantitative biochemical approaches should resolve this issue.

## Discussion

To our knowledge, *Ntl* is the first member of the SLC6/NSS family shown to provide a function essential for male fertility in any animal. *Ntl* orthologues in the Dipteran disease vectors *A. gambiae* and *A. aegypti* are highly conserved [58]. Because the pharmacology of SLC6 transporters is very highly developed for the treatment of many neurological conditions, candidate compounds that might effectively be used for vector control via male fertility through blockage of *Ntl* function may be present among compounds already screened for their effects on other NSS family members.

The most significant BLAST score for *Ntl* with a human gene product is with the human glycine transporter type 2 (GLYT2), also known as SLC6A5. This is a presynaptic glycine transporter, mutations in which are associated with autosomal recessive hyperekplexia and life threatening episodes of neonatal apnea. Individuals with mutations in SLC6A5 present with hypertonia, an exaggerated startle response to tactile or acoustic stimuli. SLC6A5 mutations result in defective subcellular localization of glycine transporter-2, decreased glycine uptake, or both, with selected mutations affecting predicted glycine and Na^+^ binding sites [59].

Since neither the germ cells nor the somatic cyst cells encasing them are known to be enervated, the *Ntl* gene product is unlikely to function as a neurotransmitter transporter. Presence of an aspartate or glycine in TM1 of a SLC6 transporter is thought to be partially responsible to distinguish between monoamine neurotransmitter transporters or amino acid transporters respectively [60]. A glycine is present between TM1a and TM1b in the *Ntl* amino acid sequence which further supports the claim for *Ntl* being an amino acid transporter. Recently, evidence has been accumulating that most of the orphan transporters of this class function as transporters of amino acids or their derivatives [20], [32]. Although amino acid uptake and reuptake are likely functions for such transporters in the gut and kidney respectively, the potential function of a male germ cell-specific transporter of this class is less clear.

The apparent reduction in glycylation of *Ntl* sperm suggests that the *Ntl* gene product is a spermatid-specific glycine transporter. This would rationalize the similarity of *Ntl* to known glycine transporters, the requirement of polyglycylation for stability and motility of microtubule-based structures in numerous species [37], [61], [62], and the previous observation that glycylation is required for male fertility in *Drosophila*. However, complete loss of glycylation results in the breakdown of the individualization complex as it translocates along the cyst [37], whereas the partial loss of glycylation suggested by these data allows efficient passage of the complex along the entire length of the 2 mm-long sperm bundle, Together, these observations argue that the structural integrity of flagellar microtubules seen in *Ntl* mutants is sufficient for sperm individualization, but not for later transitions required for transfer to the seminal vesicle and/or sperm motility, consistent with previous observations that sperm individualization and polyglycylation are tightly coordinated [38], [44], [63]. The glycylation of tubulin subunits along the inordinately long *Drosophila* sperm axonemes [64]suggests a particularly large requirement for glycine. We propose that the cytosolic pool of glycine in spermiogenic cysts is augmented by *Ntl* to accommodate this demand.

Enomoto [65] identified a human testis-specific SLC6 transporter, CT2 (aka SLC6A10), which they showed was a carnitine transporter. They also showed that the mammalian CT2 transporter is highly enriched in the epididymis, where mammalian sperm motility is activated. The immotility of *Ntl* sperm suggests a potentially parallel role for *Ntl* in the activation of *Drosophila* sperm motility. Further understanding of the mechanisms controlling the activation of sperm motility, transfer to the seminal vesicle, and how these two phenomena may be linked, might therefore be informed by future studies of *Ntl*.
